# Comparative study of titrated oral misoprostol solution and vaginal dinoprostone for labor induction at term pregnancy

**DOI:** 10.1007/s00404-015-4000-y

**Published:** 2016-01-08

**Authors:** Xiu Wang, Aijun Yang, Qingyong Ma, Xuelan Li, Li Qin, Tongqiang He

**Affiliations:** 1 Department of Gynecology and Obstetrics, Affiliated Guangren Hospital of Xi’an Jiaotong University, No. 21, Jiefang Road, Xi’an, 710004 Shaanxi China; 2 Department of Hepatobiliary Surgery, First Affiliated Hospital of Medical College, Xi’an Jiaotong University, No. 61, Jiankang Road, Xi’an, 710061 Shaanxi China; 3 Department of Gynecology and Obstetrics, First Affiliated Hospital of Medical College, Xi’an Jiaotong University, No. 61, Jiankang Road, Xi’an, 710061 Shaanxi China; 4 Obstetric Department of Shannxi Province People Hospital, No. 42, Friendship Road, Xi’an, 710068 Shaanxi China; 5 Obstetric Department of Maternal and Child Care Service Center of Northwest, No. 1616, Yanxiang Road, Xi’an, 710008 Shaanxi China

**Keywords:** Titrated oral misoprostol, Labor induction, Dinoprostone, Efficacy and safety

## Abstract

**Objective:**

To evaluate effectiveness and safety of titrated oral misoprostol solution (OMS) in comparison with vaginal dinoprostone for cervix ripening and labor induction in term pregnant women.

**Methods:**

A multicenter randomized controlled trial of women with term singleton pregnancy with indications for labor induction; 481 participants were allocated to receive titrated OMS with different doses by hourly administration according to the procedure or insert vaginal dinoprostone for cervix ripening and labor induction to compare maternal outcomes including indication of labor induction, mode of outcome of delivery, maternal morbidity, and neonatal outcomes between two groups for evaluating the efficacy and safety of titrated oral misoprostol induction.

**Result:**

Proportion of delivery within 12 h of titrated oral misoprostol is significantly less than vaginal dinoprostone (*p* = 0.03), but no difference of total vaginal delivery rate (*p* = 0.93); the mean time of first treatment to vaginal delivery was longer in OMS group (21.3 ± 14.5 h) compared with the vaginal dinoprostone group (15.7 ± 9.6 h). Although the proportion of cesarean section between the two groups showed no statistically significant difference, OMS group showed significantly lower frequency of uterine hyperstimulation, hypertonus, partus precipitatus and non-reassuring fetal heart rate than dinoprostone group. Neonatal outcomes were similar evaluating from Apgar score and NICU admission. Our study also showed that labor induction of women with cervix Bishop score ≤3 needed increased dosage of misoprostol solution.

**Conclusion:**

Titrated OMS is as effective as vaginal dinoprostone in labor induction for term pregnant women, with safer effect for its lower rate of adverse effect for women.

## Background

Induction of labor is a commonly practiced obstetric intervention designed to artificially initiate the process of effacement of cervix, dilatation and eventually delivery of baby. Adopting safe and effective methods of labor induction at appropriate gestation age can greatly decrease complications and morbidity of pregnancy and fetus. In recent years, the rate of induction presents gradually increasing tendency, and the incidence for labor induction dramatically varies 8–44 % [1–3]. Therefore, looking for induction methods with safety, efficacy, feasibility, low cost, and patient preferences, for a long time, is a pursuit of the goal of all obstetric providers.

Recently, many studies reported oral misoprostol for labor induction with different doses and interval times; at the same time, respectively compared with other induction methods including vaginal insert misoprostol, oxytocin, Foley catheter induction, oxytocin associated Foley catheter and vaginal insert Dinoprostone. The target of all these studies is to evaluate the safety and effectiveness of oral misoprostol for labor induction. According to these study results, we propose the concept that misoprostol will be a new promising agent for cervical ripening and induction of labor, but ideal dose, route and administration frequency are still needed for investigation.

Misoprostol, a synthetic prostaglandin E_1_ analog, can be administered orally, sublingually, buccally, intra-vaginally, or rectally and is used for both cervical ripening and labor induction. The World Health Organization recommends a fixed oral misoprostol dose of 25 mg every 2 h for labor induction based on moderate-quality evidence and strong recommendation [4]. However, trials continue in efforts to identify the optimum treatment regimen.

On the basis of the misoprostol pharmacokinetics, this medicine shows the characteristics of rapid oral absorption, its active metabolites of misoprostol acid in plasma reach peak value after 15 min of oral administration, and its mean tmax concentration is 0.309 μg/L. Its half-life is 20–40 min following oral administration, followed by a rapid decline to low levels during the period of 120 min, thereafter with a more gradual decline, and no drug accumulation phenomenon. According to its pharmacokinetics property, we designed the administration procedure of hourly titrated oral misoprostol dosing and gradually increasing dosage, compared with 2 h dosing; this procedure may make drug serum level more steady with better efficacy, and at last improve clinical induction outcome.

## Materials and methods

### Design

This trial was a multicenter, open-label, randomized controlled trial. The study was performed within four obstetric centers; participating hospitals can be district, teaching or academic hospitals. Before entry into the study, women were informed about the aims, methods, reasonably anticipated benefits and potential hazards of the study. Prior to interview, informed consents had been taken from every respondent. Patients who met the selection criteria were explained about advantages and disadvantages of the procedure. Among them, those who provided their informed consents were interviewed and recruited in the study. They were informed that their participation would been voluntary and may withdraw consent for participation at any time during the study.

### Inclusion criteria

All the women must be singleton pregnancies with occipital presentation, nullipara, gestational age is at least 36 weeks, Bishop score less than six, no vaginal delivery contraindication, e.g. cephalopelvic disproportion, mal-presentation, fetal compromise, no reassuring fetal heart rate pattern, previous scar and antepartum haemorrhage.

### Exclusion criteria

Women with any contraindication to induction and vagina delivery, allergic to prostaglandin, and with complication of glaucoma, asthma and allergic colitis, woman with heart, liver, renal and adrenal cortex insufficiency.

### Multicenter randomized control design

This multicenter randomized controlled trial was conducted from January to October in 2014 at four gynecologic and obstetric departments (including Obstetric Department of Affiliated Guangren Hospital of Xi’an Jiaotong University; Obstetric Department of First Affiliated Hospital of Medical College of Xi’an Jiaotong University in China; Obstetric Department of Maternal and Child Care Service Center of Northwest; Obstetric Department of Shannxi Province People Hospital). On enrollment, an opaque envelope corresponding to the participant’s enrollment number was opened assigning women to either oral misoprostol or vaginal insert dinoprostone group determined by a computer-generated randomization sequence.

### Methods of administration and evaluation

Based on the WHO labor induction recommendation, and for the purpose of achieving precise oral misoprostol dosage, we pulverized one misoprostol tablet (200 μg), dissolved into 200 ml water, then one misoprostol tablet (200 μg) was made into 1 μg/ml concentration oral misoprostol solution (OMS), and preserved at room temperature for 24 h.

**Fig. 1:**
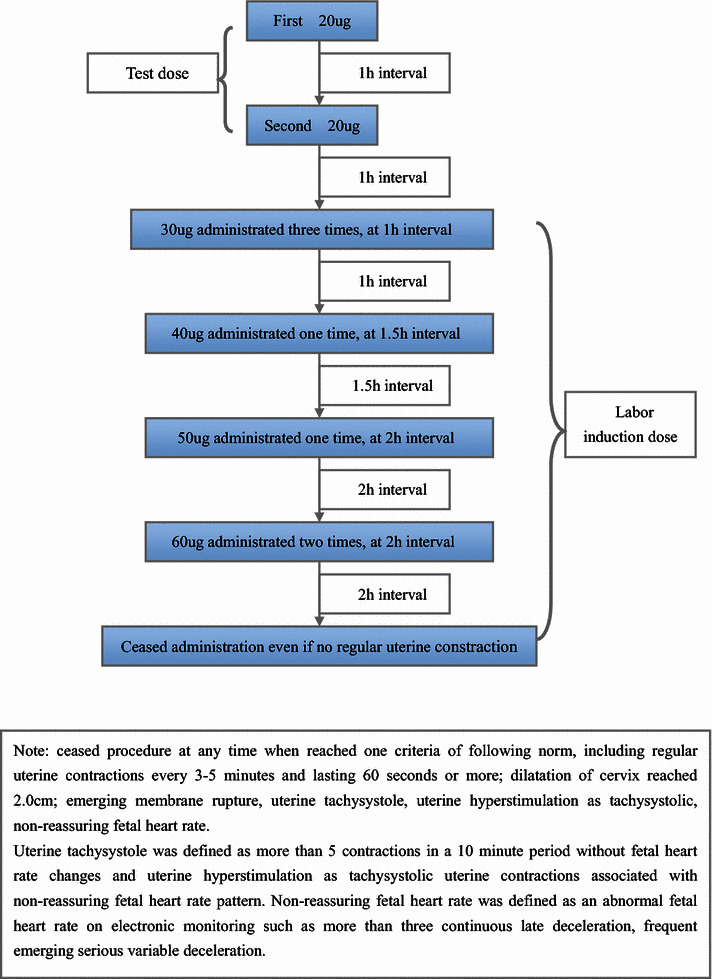
Flow diagram of process of hourly adminstration titrated oral misoprostol solution and ceased procedure criteria

All the women enrolled into OMS group were given misoprostol solution according to the procedure (Fig. 1) and ceased procedure at any time when reached one of the following criteria: including regular uterine contractions every 3–5 min and lasting 60 s or more; dilatation of cervix reached 2.0 cm; emerging membrane rupture; uterine tachysystole; uterine hyperstimulation as tachysystolic, non-reassuring fetal heart rate. The whole procedure spent 10.5 h, when woman ends the first procedure with no signs of regular uterine contraction, after 6 h interval, we would begun second cycles procedure, in second procedure, the criteria of ceasing procedure is as same as previous norms.

The vaginal insert dinoprostone was given according to the drug protocol. Dinoprostone should be removed from the freezer in direct connection with the insertion, and be inserted high into the posterior vaginal fornix using only small amount of water soluble lubricants to aid insertion. After dinoprostone has been inserted, the withdrawal tap may be cut with scissors ensuring there is sufficient tap outside the vagina to allow removal. The norms of terminating drug administration were as same as OMS group.

When participants began the process of labor induction, abdominal palpation uterine contraction and electric monitoring would be taken to evaluate uterine contraction, fetal heart-rate every hour, and vaginal exam to know the dilatation of cervix every 1–2 h.

### Outcomes measure

General situation of participants: including age, gestational age, body mass index (BMI). Indication of labor induction: premature rupture of membrane (PROM), oligohydramnios, post-term gestation, pre-eclampsia, gestational diabetes mellitus (GDM) controlled without insulin, and other reasons. Mode and outcome of delivery: including mode of delivery such as spontaneous delivery, vaginal operative delivery or cesarean section, and the reason for operative delivery and cesarean section; Induction to delivery time: less than 12 h delivery, 12–24 h delivery and 24–48 h delivery; Cervix Bishop score; total oral misoprostol dosage; oxytocin use situation; requirement of analgetics; pertus precipitatus (defined as the total time of labor stage less than 3 h); reasons of cesarean section and operative delivery. Maternal morbidity: postpartum blood transfusion and number of packed cell, Tachysystole (defined as more than five contractions in 10 min); hyperstimulation (defined as tachysystole with FHR changes); uterine hypertonus (defined as a contraction lasting longer than 2 min with FHR changes); Uterine rupture (occurrence of clinical symptoms include abdominal pain, abnormal fetal heart rate pattern, acute loss of contractions and vaginal blood loss) leading to an emergency cesarean delivery, at which the presumed diagnosis of uterine rupture was confirmed; or peripartum hysterectomy or laparotomy for uterine rupture after vaginal birth); Maternal infection during labor (defined as fever, i.e. temperature ≥37.8 °C, or fetal tachycardia and start of antibiotics); maternal infection within 1 week postpartum (defined as fever, i.e. temperature ≥37.8 °C, and start of antibiotics); start of intravenous antibiotics; endo (myo)metritis or urinary tract infection within 1 week postpartum (proven positive vaginal/urine culture); other medication used during labor such as tocolytics. Neonatal parameters consisting of: fetal tachycardia (sustained fetal heart rate above 160 beats per minute), fetal distress; weight at birth; meconium-stained liquor; Apgar scores <7 at 1, 5, and 10 min; admission to the neonatal ward/NICU and its reason (suspected infection, infection proven by positive culture, other reason admission to medium or intensive care).

### Statistical analysis

All data were analyzed by statistics software SPSS 19.0. Results were given as mean ± SD or percentage, time intervals were analyzed with ANOVA test, other data were analyzed with *χ* ^2^ for qualitative and Student’s *t* test for quantitative variables. All tests were two-sided, *p* < 0.05 was considered statistically significant.

## Results

**Fig. 2:**
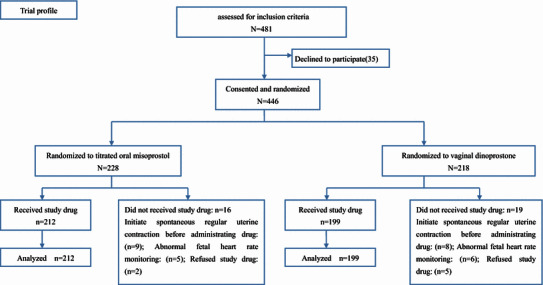
Procedures for the selection and follow-up of participants

A total of 481 women met the inclusion criteria and approached regarding study participation, 35 women declined, of whom 228 women were randomized to OMS group and 218 women to vaginal dinoprostone group. 35 participants did not receive the assigned drug, 16 women in the OMS group and 19 women in vaginal dinoprostone group. Reasons for not receiving the study drug mainly included the following causes: initiating spontaneous regular uterine contraction, non-reassuring FHR, and refusal of study drug (Fig. 2).

There were no differences in maternal age, gestational age and body weight index between OMS and vagina dinoprostone groups (Table 1). We also compared the labor induction indication between two groups, showed no difference of indication in oligohydramnios, posterm gestation, gestational diabetes, preterm rupture of membrane, pre-eclampsia, and fetal growth restriction (Table 2).

**Table 1:** General condition of study participants

	Oral misoprostol (*n* = 212)	Dinoprostone (*n* = 199)	*p* value
Maternal age (years)	27.8 ± 4.8 (18–39)	28.3 ± 5.6 (18–35)	0.84
Gestational age (weeks)	39.5 ± 6.2 (36–42)	38.8 ± 7.1 (36–42)	0.98
BMI (Kg/m^2^)	25.3 ± 8.7 (18.9–33.5)	26.1 ± 8.2 (18.5–32.8)	0.89

Data are mean ± SD (range)

*BMI* body mass index

**Table 2:** Indication of labor induction

	Oral misoprostol (*n* = 212)	Dinoprostone (*n* = 199)	*p* value
Oligohydramnios	127 (56.9)	117 (58.7)	0.92
Post term-gestation	32 (15.1)	36 (18.0)	0.87
GDM	13 (6.1)	11 (5.5)	0.72
PROM	19 (8.9)	15 (7.5)	0.82
Pre-eclampsia	12 (5.6)	10 (5.0)	0.90
FGR	6 (2.8)	6 (3.0)	0.91
Other	3 (1.4)	4 (2.0)	0.73

Data are number (percentage)

*GDM* gestational diabetes mellitus, *PROM* premature rupture of membrane, *FGR* fetal growth restriction

Proportion of vaginal delivery between OMS (182/212, 85.8 %) and vaginal dinoprostone (163/199, 81.9 %) showed no difference. We further analyzed from the interval of time, within 12 h the proportion of vaginal delivery in OMS was lower than dinoprostone group with significant difference (*p* = 0.03), but during 24–48 h the percentage of vaginal delivery was obviously higher than dinoprostone (*p* = 0.04), and during 12–24 h delivery proportion was similar between two groups (*p* = 0.89); however, the mean time of first treatment to vaginal delivery was significantly longer in OMS group (21.3 ± 14.5 h) compared with the vaginal dinoprostone group (15.7 ± 9.6 h). Proportion of cesarean section in vaginal dinoprostone (36/199, 18.0 %) was a little higher than OMS (30/212, 14.2 %), but statistically no significance, analyzing the indication of cesarean section, the dinoprostone group showed higher fetal distress, but the difference without statistically significant, and proportions of delivery process block were similar between groups. The proportion of total vaginal operative delivery showed no significant difference between the two groups, and causes of vaginal operative delivery (forceps and vacuum extractor delivery) mainly include uterine atony, fetal distress and delivery process block, proportion of three main causes of vaginal operative delivery were similar. Compared other respects include using oxytocin and requirement of analgetics showed no difference between groups. Proportion of partus precipitatus in vaginal dinoprostone (9/163, 5.5 %) was higher than oral misoprostol (5/182, 2.7 %) (Table 3).

**Table 3:** Mode and outcome of delivery

	Oral misoprostol (*n* = 212)	Dinoprostone (*n* = 199)	*p* value
Delivered vaginally	182/212 (85.8)	163/199 (81.9)	0.93
<12 h	39/182 (21.4)	65/163 (40.1)	0.03
During 12–24 h	79/182 (43.4)	66/163 (40.4)	0.89
During 24–48 h	58/182 (31.9)	28/163 (17.2)	0.04
First treatment to vaginal delivery, mean *h*	21.3 ± 14.5	15.7 ± 9.6	0.04
Bishop score			
≤ 3	69/182 (37.9)	57/163 (34.9)	0.32
4–6	113/182 (62.1)	106/163 (65.0)	0.78
Cesarean section	30/212 (14.2)	36/199 (18.0)	0.34
Fetal distress	7/30 (23.3)	10/36 (27.8)	0.44
Delivery process block	23/30 (76.7)	26/36 (72.2)	0.86
Vaginal operative delivery	22/182 (12.1)	29/163 (17.8)	0.50
Uterine atony	8/22 (36.4)	9/29 (31.0)	0.80
Fetal distress	8/22 (36.4)	12/29 (41.4)	0.79
Delivery process block	6/22 (27.2)	8/29 (27.6)	1.00
Oxytocin use	32/182 (17.6)	28/163 (17.1)	1.00
Requirement of analgesia	92/182 (50.5)	75/163 (46.0)	0.92
Partus precipitatus	5/182 (2.7)	9/163 (5.5)	0.04

Data are mean ± SD (range) or number (percentage)

Based on women cervix Bishop score, we divided into two subgroups, respectively, ≤3 and 4–6. We found that proportions of successful induction were similar between groups, however, Bishop score less than 3 or 4–6, but women with Bishop score ≤3 in oral misoprostol group administered more misoprostol solution dose. The mean dosage of oral misoprostol solution is 180 ± 120 μg, and further stratified by cervix Bishop score, the mean dosage of misoprostol 435 ± 124 μg in the Bishop score ≤3, and 152 ± 95 μg in the Bishop score 4–6 subgroup. Duration time of first treatment to vaginal delivery was longer than Bishop score 4–6 subgroup; 51 women gave birth during 24–48 h. We also compared the frequency of maternal adverse events between groups, the overall uterine tachysystole was not different between the two groups; 18 women in vaginal dinoprostone group had hyperstimulation, its frequency is significantly higher than oral misoprostol group (*p* = 0.03). Five women in oral misoprostol and 16 women in vaginal dinoprostone presented uterine hypertonus with significantly difference (*p* = 0.03). 6 women in OMS and 17 in dinoprostone group needed tocolytics to inhibit uterine hyperstimulation, tachysystole and hypertonus, the result showed significantly difference (*p* = 0.04). The proportion of membrane rupture phenomenon in OMS (59/212, 27.8 %) is higher than vaginal dinoprostone (26/199, 13.1 %). Proportion of postpartum heamorrhage and intravenous antibiotics was similar between the groups, and maternal other adverse event including fever, shivering, nausea and vomiting also present no difference (Table 4).

**Table 4:** Maternal morbidity

	Oral misoprostol (*n* = 212)	Dinoprostone (*n* = 199)	*p* value
Tachysystole	15/212 (7.0)	19/199 (9.5)	0.61
Hyperstimulation	5/212 (2.4)	18/199 (9.0)	0.03
Uterine hypertonus	5/212 (2.4)	16/199 (8.0)	0.03
Uterine rupture	0/212	0/199	
membrane rupture	59/212 (27.8)	26/199 (13.1)	0.02
Tocolytics	6/212 (2.8)	17/199 (8.5)	0.04
Postpartum heamorrhage (ml)	14/212 (6.6)	15/199 (7.5)	0.73
≥500	10/14 (71.4)	10/15 (66.7)	
≥1000	4/14 (28.6)	5/15 (33.3)	
Intravenous antibiotics	32/212 (15.1)	35/199 (17.6)	0.96
Fever	2	0	
Shivering	1	1	
Nausea and vomiting	2	1	

Data are number (percentage)

Neonatal outcomes are shown in Table 5, neonatal Apgar scores ≤7 at the interval of 1, 5 and 10 min were no different, and the proportions of Meconium-stained liquor and neonate NICU admission were also similar, but non-reassuring fetal heart rate frequency in vaginal dinoprostone is obviously higher than OMS group (*p* = 0.04).

**Table 5:** Neonatal outcomes

	Oral misoprostol (*n* = 212)	Dinoprostone (*n* = 199)	*p* value
Weight (g)	3020 ± 566.5	3110 ± 499.3	1.0
Non-reassuring fetal heart rate	15/212 (7.1)	22/199 (11.1)	0.04
Meconium-stained liquor	46/212 (21.7)	55/199 (27.6)	0.58
Apgar score			
≤7 at 1 min	8/212 (3.8)	9/199 (4.5)	0.66
≤7 at 5 min	3/212 (1.4)	3/199 (1.5)	1.0
≤7 at 10 min	0	1	
NICU admission	15/212 (7.0)	15/199 (7.5)	1.0
Infant death	0	0	

Data are number (percentage)

## Discussion

Labor inductions have increased steadily over the past two decades, meanwhile many methods have been tested, but prostaglandins remain a preferred method for cervical ripening and labor induction [5, 6]. In 2001, Hofmeyr [7] compared the titrated oral misoprostol solution with vaginal dinoprostone for labor induction, proposed the new approach, titrated oral misoprostol solution administration and was successful in minimizing the risk of uterine hyperstimulation, and with no significant difference in maternal adverse effect and neonatal outcome between the two groups. From then on, many obstetricians pay attention to titrated oral misoprostol induction method because of its greater acceptance and fewer adverse effects. To further evaluate safety and efficacy of titrate oral misoprostol induction, many obstetricians compared with different labor induction method. Comparing OMS with conventional oxytocin induction, the result showed misoprostol was a safe and effective drug with low complications for the induction of labor, failure was seen less with misoprostol and cesarean sections are less frequently indicated as compared to oxytocin [8–10]. Comparing OMS with Foley catheter in cervical ripening and induction effect, OMS group showed higher rate of delivery in 24 h, and in labor augmentation, cesarean section and instrumental delivery were somewhat fewer frequency than Foley group, but these differences were not statistically significant, side effects and neonatal complications were similar between the two groups [11]. A comparative study about oral and vaginal misoprostol for labor induction, which showed oral misoprostol was as effective as vaginal misoprostol with the advantage of shorter induction delivery interval, lower cesarean section rate, and lower incidence of failed induction rate, lower proportion of fetal distress and easy intake [12, 13]. In 2011, WHO guideline also strongly recommended oral misoprostol (25 μg, 2-hourly) than vaginal low-dose misoprostol (25 μg, 6 hourly) for induction of labor. A randomized controlled trial about comparison of OMS with vaginal misoprostol showed that OMS associated with a lower incidence of uterine hyperstimulation and a lower cesarean delivery rate than vaginal misoprostol for labor induction in patients with unfavorable cervix [14]. A randomized double-blind trial compared efficacy and safety about OMS and oral misoprostol, participants were allocated to receive 20 ml of misoprostol solution (1 μg/ml) orally every 1 h for four doses then titrated to 40 μg every 1 h (OMS group) or 50 μg of misoprostol orally every 4 h up to 12 h (oral group), and concluded that oral misoprostol was as effective as titrated misoprostol for cervical ripening and labor induction, but had a lower incidence of tachysystole and a lower total dose of misoprostol are required. Its results seem to be contradictory to previous studies, perhaps OMS dose and administration interval time difference could affect the outcomes of induction of labor [15]. A systematic review and net meta-analysis based on the data of Cochrane Pregnancy and Childbirth Group’s Database of Trials show low dose (<50 µg) titrated oral misoprostol solution had the lowest probability of cesarean section, whereas vaginal misprostol (≥50 µg) had the highest probability of achieving a vaginal delivery within 24 h [6].

However, up to now, there are still no definite conclusions about the optimal dose, interval time and route of administration. In our study, we take the new method that gradually increase titrated oral misoprostol solution dose, and adminstrate by 1–2 h, compare with vaginal dinoprotone for evaluating its effectiveness and safety. Evaluation from mode and outcomes of delivery, which is similar for total proportion of vaginal delivery between the two groups during 48 h, nevertheless titrated oral misoprostol has the property of relatively slower duration of labor compared with dinoprostone, but accompanied the lower rate of uterine hyperstimulation, hypertonus, usage frequency of tocolytics and non-reassuring fetal heart; and concurrently OMS with lower incidence of tachysystole, cesarean delivery rate although with little significant difference in our study. Analyzed from the respect of neonatal outcomes, meconium-stained liquor rate in OMS group are lower than vaginal dinoprostone. From these points of view, titrated oral misoprostol is safer than dinoprostone for women labor induction, these results are as same as other studies.

The incidence of non-reassuring fetal heart rate in dinoprostone was significantly higher than OMS group, we also found that the rate of fetal distress in dinoprotone was also higher than OMS group, but the total proportion of cesarean section between two group showed no difference because of higher incidence of usage of tocolytics in dinoprostone. Uterine tachysystole, hyperstimulation, hypertonus accompanied non-reassuring fetal heart rate, tocolytics should be used to at once, so that a part of women would not been dealt with cesarean section. I think that may be the reason of our study with higher non-reassuring fetal heart rate rather without higher incidence of cesarean section in dinoprostone.

The phenomenon of higher proportion membrane rupture in oral misoprostol perplexed us, and we exclude the reason of amniotic membrane infection by placental histopathology. This phenomenon was not reported in other document literature, but 54 women in OMS group and 23 women in dinoprostone group presented on regular uterine contraction and delivered within 12 h, and three women did not present regular uterine contraction after 12 h, then experienced oxytocin induction and delivered, two women who presented delivery process block were transferred to cesarean section. We thought that higher incidence of membrane rupture in OMS group needed to be further studied by expanded sample size.

Based on the cervix Bishop score, we found that with the lower of cervix Bishop score, corresponding emergency needed higher dose of oral misoprostol solution and longer duration of time for labor induction. We consider that other procedure of labor induction with OMS will be studied, for women with lower Bishop score ≤3, could be administrated higher dose of initial dose, and increased more than 10 µg every time, and been longer than 2 h interval. Of course, optimum dose and interval of administration for women with low Bishop score need further extensive and intensive study.

In conclusion, titrated oral misoprostol given hourly for labor induction is as effective as vaginal dinoprostone from the respect of maternal/fetal outcomes, but titrated oral misoprostol is associated with lower rate of uterine hyperstimulation, hypertonus, tachysystole, and nonreassuring fetal heart compared with vaginal dinoprostone. Taking into account the higher vaginal delivery rate, lower adverse effect for maternal/fetal outcomes, costs and women’s preferences; our study consider that titrated oral misoprostol solution would be a safe, cost-effective and patient favorite way for inducing labor.
